# Penile shaft sinus: A sequalae of circumcision in urethral duplication

**DOI:** 10.4103/0970-1591.45554

**Published:** 2009

**Authors:** Lukman O. Abdur-Rahman, AbdulRasheed A, Nasir John O, Agboola, James O. Adeniran

**Affiliations:** Paediatric Surgery Unit, University of Ilorin Teaching Hospital, Ilorin, P. O. Box 5291, Ilorin, Ilorin-240 001, Kwara, Nigeria

**Keywords:** Circumcision, penile sinus, urethral duplication

## Abstract

Urethral duplication (UD) is rare congenital anomalies with varied presentation. Careful clinical evaluation of children by specialist would enhance diagnosis, adequate management and reduce occurrence of complication. We present a 12-year-old boy with chronic post circumcision ventral penile sinus that was successfully managed for urethral duplication.

## INTRODUCTION

Urethral duplication (UD) is a rare congenital anomaly with varied clinical presentations and treatment. The embryologic development of UD is still poorly understood and theories have shown that it is due to cloaca membrane/genital tubercle and urogenital sinus anomalies.[1]

All patients should be thoroughly evaluated because of strong association with other congenital anomalies and significant surgical management challenge this may present which may lead to unpredictable outcome.[2,3]

We report a case of incomplete UD in a 12-year-old boy who presented with a post-circumcision penile sinus.

## CASE REPORT

**Figure 1:**
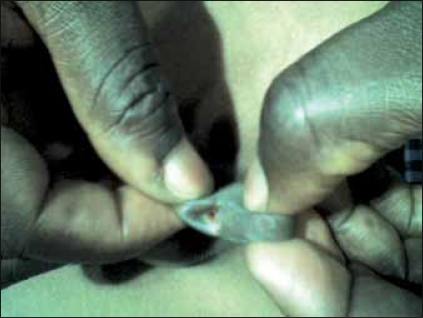
Penile sinus on the ventral surface of penile. note normal meatus on the glans

**Figure 2:**
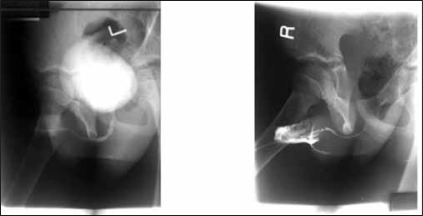
Micturating cystourethrogram and penile sinugram

**Figure 3:**
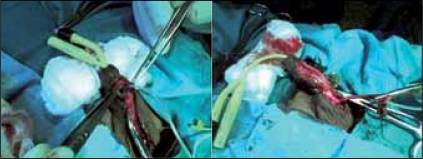
Tube probe in the duplicated urethra

**Figure 4:**
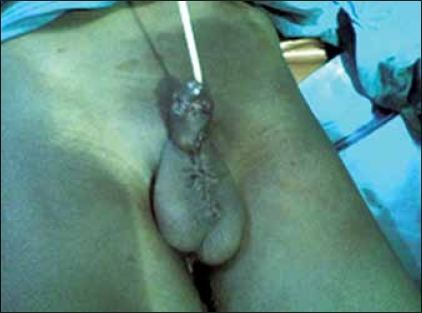
Post operative photograph with urethral catheter *in-situ*

A 12-year-old boy who presented with recurrent sero-mucoid discharge from a wound on the ventral surface of the penis. This occurred post circumcision done at home by a ‘nurse’ on the sixth day of live because of a distal penile swelling. Pregnancy and delivery were uneventful. The wound refused to heal in spite of repeated dressings and use of antibiotics. He had good urinary stream and normal developmental milestone. No history of recurrent urinary tract infections. He was a healthy looking young boy with adequate penile size; there was an ulcer with a background sinus and serous fluid discharge from it [Figure 1]. The urethral meatus was normally sited and adequate in size. A diagnosis of penile sinus to rule out urethral duplication was entertained. He had a micturating cystourethrogram [Figure 2] which showed normal bladder and single urethral channel. A penile sinogram showed a shallow tract that is not communicating with the orthotopic urethral. Examination under anaesthesia using a lubricated size 6 French gauge feeding tube passed via the sinus went through a long tract up to the floor of the pelvis but ended blindly [Figure 3]. This tract coursed initially in the ventral surface but later to the right lateral aspect of the orthotopic urethral from midshaft to the bulbar urethra. The whole tract was excised and histology confirmed an urothelium. Postoperative period was uneventful and patient has remained well [Figure 4].

## DISCUSSION

The occurrence of UD presents a great challenge in its identification and management because of its rarity and its variants.[4,5] A little over 200 cases have been published world wide including a case reported by Okeke *et al.*[6] from Nigeria. Innes Williams[7] described a morphologic classification in which urethral duplication could be; epispadial UD, hypospadial UD, spindle urethras, bifid urethras with accessory preanal branch and collateral duplication and Effmann *et al.*[8] classification described the variants of complete and incomplete UD with proximal, distal or non communicating orthotopic urethral. Our patient fits into the Innes William's bifid urethras with accessory preanal branch and Effmann's incomplete type IA UD.

In spite of warnings to practitioners against circumcision in cases of congenital abnormalities of the penis especially in cases of hypospadias and epispadias, this patient had his circumcision done within the first week of live because of the abnormal swelling noticed on the ventrum of the distal penile /preputial skin which left a residual sinus. In our environment, circumcision practice is by the untrained (traditional circumcisionists) and junior cadre health workers usually in late childhood between 5 and 12 years when children are gathered in groups for them to be initiated into manhood.

The differential diagnoses are congenital anterior urethrocutaneous fistula,[9] excised urethral diverticulum from urethral valve and post circumcision urethrocutaneous fistula. The micturating cystourethrogram and penile sinogram done outlined separate channels between the orthotopic urethra and the duplicate urethra however, it was shallow and did not get to the depth of the tract which was later demonstrated at operation by a probe. This buttress the fact that infusion of contrast under pressure and fluoroscopy (not available in our centre) including examination under anaesthsia would assist in demonstrating the tract.

The danger in poor demonstration of tract is the possibility of inadequate and improper preparation for surgery leaving a residual tract or damage to adjoining tissues. Paediatric urethrocystoscopy for better definition of the UD was not available at our centre. Antibiotic and wound dressing treatment are ineffective, and other treatment such as diathermocoagulation or the injection of caustic substance into the accessory duct have been condemned and abandoned.[5] The surgical treatment would depend on the type of urethral duplication and associated malformation. All effort should be made to preserve the sphincter.

In conclusion, urethral duplication, though rare, have good prognosis if adequately managed. Pre-emptive circumcision should be avoided in patients with any penile malformation and patient should be referred to a trained surgeon.
